# The Feasibility and Efficiency of Remote Spirometry System on the Pulmonary Function for Multiple Ribs Fracture Patients

**DOI:** 10.3390/jpm11111067

**Published:** 2021-10-23

**Authors:** Chien-An Liao, Tai-Horng Young, Chi-Tung Cheng, Ling-Wei Kuo, Chih-Yuan Fu, Chih-Po Hsu, Szu-An Chen, Yu-San Tee, Chih-Hsun Hsieh, Chih-Chi Chen, Chien-Hung Liao

**Affiliations:** 1 Institute of Biomedical Engineering, College of Medicine and College of Engineering, National Taiwan University, Taipei 10547, Taiwan; victorgoer@gmail.com (C.-A.L.); thyung@ntu.edu.tw (T.-H.Y.); 2 Department of Trauma and Emergency Surgery, Chang Gung Memorial Hospital, Chang Gung University, 5, Fu-Hsing Street, Kwei Shan Township, Taoyuan 33328, Taiwan; atong89130@gmail.com (C.-T.C.); m0102@cgmh.org.tw (L.-W.K.); drfu5564@gmail.com (C.-Y.F.); m7831@cgmh.org.tw (C.-P.H.); wacamama@cgmh.org.tw (S.-A.C.); b9402011@cgmh.org.tw (Y.-S.T.); hsiehchihsun@yahoo.com.tw (C.-H.H.); 3 Department of Physical Medicine and Rehabilitation, Chang Gung Memorial Hospital, Chang Gung University, 5, Fu-Hsing Street, Kwei Shan Township, Taoyuan 33328, Taiwan

**Keywords:** multiple rib fractures, lung function, remote spirometry system

## Abstract

Background: Multiple rib fractures is a common chest trauma with a significant and sustained impact on pulmonary function and quality of life. Continuous monitoring of the pulmonary function parameter was necessary to adjust the therapeutic goals in these patients. We developed an internet-based remote system for lung function monitoring with a remote spirometry and smart device application to follow up these patients consecutively. Method: From Jan 2021 to April 2021, we conducted a prospective study that applied an intelligent spirometry system for patients with multiple rib fractures. With informed consent, we collected clinical data from them and introduced the remote spirometry system. We followed up with these patients for 12 weeks after trauma and compared the recovery of pulmonary function parameters and clinical outcomes. Result: A total of 21 patients were enrolled in our study. We divided them into two groups by the compliance to this remote spirometry system. The improvement of forced vital capacity was better in the good compliance group than the poor compliance group (110% versus 21%, *p* value 0.049). Moreover, the complication rate was also lower in the good compliance group than the poor compliance group (10% versus 66.7% *p* value 0.017). Conclusions: Remote spirometry system is a novel system that can help in lung rehabilitation in patients with multiple rib fractures. Patients that cooperate well with this system presented superior lung function improvement and inferior complication rate.

## 1. Introduction

The rib fracture is one of the most common chest traumas and has affected millions of patients worldwide. Significant pain, thoracic cavity limitation, and respiratory distress impact the patient’s daily quality of life [1,2,3,4]. At the most severe end of the spectrum of rib fracture injuries, flail chest is associated with life-threatening injuries and significant mortality [5,6]. With the advance of knowledge and therapeutic options, the prognosis and outcome improved. Aside from the acute impact of rib fracture injury, more prolonged term morbidity of pain, disability, and deformity have been described [7,8]. Multiple rib fractures have been shown to have a significant and sustained impact on pulmonary function. Prolonged pulmonary limitation and pain may persist for months. However, we did not have proper tools to follow up on this type of patient.

For monitoring, self-report questionnaires are the most common method for pain and analgesia assessment, and they have the advantage of cost-effectiveness and ease of administration [9]. However, the potential disadvantages of self-report questionnaires are that they may be less reliable and may also be affected by external factors, such as social desirability, age, the complexity of the questionnaire, and the participants’ recall ability [10,11]. The current pulmonary evaluation protocol is a scheduled pulmonary function test (PFT) by spirometry. However, the patient cannot report their data daily and needs to go to the institute with close contact with healthcare providers. In the pandemic era, an alternative method should be applied to assist this type of patient [12].

Telemedicine has begun to supplement in the last decade, and applications for the healthcare sector have been enriched by numerous innovations such as weight reduction, depression, or diabetes [13,14]. Existing platforms allowed virtual follow-up after elective surgery. A randomized clinical trial by Denis et al. [15] investigated the outcome of lung cancer patients. It showed a significantly better survival for patients (median overall survival 19 vs. 12 months) using a web-based tool for periodical documentation of symptoms and side effects during follow-up. Several studies have demonstrated that mHealth technology has improved clinical outcomes in medical patients by improving the control of cardiac function and glycemic hemostasis, enhancing medication compliance, and shortening of hospital stay [16,17,18]. These platforms have been met with wide acceptance and enthusiasm by patients and their surgeons in the low-risk, elective surgery cohorts studied. Although the experience of using the mHealth app in trauma care is limited, it is suggested that trauma patients can benefit from this new technology and restore the critical decline in physical and medical functions.

We developed an internet-based remote protocol for pulmonary monitoring with a remote spirometry and an app. We conducted a prospective pilot study to investigate the adequacy of the intelligent respiratory monitoring spirometry and app that can help patients and caregivers to manage their pulmonary rehabilitation at home properly. This study focused on the feasibility and clinical value of the remote spirometry in patients with rib fractures. In addition, this study determined the investigation of attitudes and expectations toward user satisfaction, program adherence (intervention use), and willingness to participate.

This study mainly contains three different sections described as follows: The first is defining the study patients and how we categorized them. We also introduced our clinical targets that are going to be analyzed in the first section. The second part is the clinical outcome and the result of this remote spirometry system. The final section is the discussion of our results. Conclusions and outlook are also in this section.

## 2. Materials and Methods

### Study Population

This is a prospective pilot study to evaluate the feasibility and clinical value of an app-based intelligent spirometry for multiple rib fractures patients. In this study, eligible participants were adult inpatients (aged ≥ 20 years) on the acute care surgery service of a trauma center. We enrolled patients who suffered from rib fractures and received treatment at our institution from Jan 2021 to April 2021. We included all patients suffering from multiple traumatic rib fractures of more than three ribs. This study protocol was approved by the institutional review board of the Chang Gung Memorial Hospital (201900495B0).

**Figure 1:**
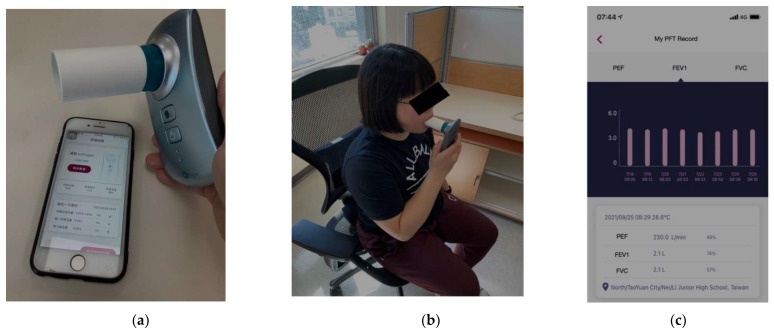
The architecture and the application of Restart system: (**a**) the remote spirometry and the app system on the smart device; (**b**) the participant performing the remote spirometry; (**c**) the presentation of pulmonary function parameters of the patients using Restart system.

We used the Restart system to monitor the respiratory parameters of rib fracture patients. This system is composed of one remote spirometry, ezOxygen, and Healthy Lung app. The remote spirometry, which uses passive ultrasonic detection to determine the airflow rate, is used as standard spirometry to measure the forced vital capacity (FVC), peak expiratory flow (PEF), and the forced expiratory volume (FEV1) for participants. The signal and data will synchronize in the Healthy Lung app, an iOS/Android app that facilitates patients who have received spirometry data and record the clinical variables (associated discomfort and analgesic ingestion), and transmit digital data to the medical staff. This app was developed and modified by healthcare professionals and software programmers to fulfill the needs of the patients. Figure 1 presents the system architecture.

We excluded the patients presenting hemodynamic instability at arrival who needed resuscitation, intubation and ventilator necessary. We also excluded Glasgow Coma scale (GCS) < 13 and severe facial bone fracture or airway injuries that caused patients unable to perform spirometry. Notably, patients were excluded if they had neurologic or cognitive disorders prohibiting their usage of the spirometry and app. Patients or caregivers without a smartphone or tablet were also excluded from this study. All subjects who met inclusion criteria were approached to participate and informed consent was obtained. The research assistant visited the participants in less than 24 h to record the acute phase lung function. Before the enrollment of this study, the research assistant evaluated the patients’ familiarity with wearable devices and smartphones. If the patients were not confident about using these devices, we provided further instruction to their caregivers. As Figure 1 presented, once the patients began to use the spirometry they could read their PFT parameters immediately on the app and the input was synchronized to the mobile device when internet access was available and the smartphones delivered the information to the server.

All the PFT parameters were sent back to us either in hospital or at home for collection. This function of interservice data transmission worked well with no abnormal events reported by the patients. The assistant kept visiting the patient for further training and to observe the usage of the Restart system. Patients used the remote spirometry at least once per day in the hospital and back at home. They were requested to continue to perform PFT until 8 weeks after discharge. We suggested our patients perform the spirometry in the early morning after having breakfast every day. Patients should perform spirometry at least once a day but they were not limited to that frequency. All the data will be recorded in our system. All the patients also have the same clinical follow-up interval which was 2, 6, and 12 weeks after discharge. Plain posteroanterior chest X-ray (CXR) was arranged in each outpatient clinic visit for an evaluation of possible residual hemothorax or pleural effusion. There was another medical staff who did not participate in designing this system who independently reviewed and analyzed the data.

We formally tested the usability and feasibility of the Restart system for patients with rib fractures at a major academic trauma center. The app was loaded onto the smartphone in the iOS or Android system. We assessed patients’ baseline familiarity with smartphones prior to testing. User tasks included: waking up the device, launching the app, information input, beginning respiration with a spirometry, review and retake or acceptance of captured data, question response, and submission. The completeness of the steps was recorded. We also monitor the frequency of usage of the Restart system of the patients with rib fractures to evaluate the compliance of the remote system to monitor the patients. We define poor compliance as less than 3 times of system usage in a week or no PFT data input for more than 10 days.

The pulmonary parameters as the FVC, FEV1, and PEF for participants were recorded and analyzed. The outcome of clinical value was to validate the usage of intelligent systems that can reduce the analgesic usage, the recovered PFT, the result of residual amount of pleural effusion, the complication, and the incidence of emergency or clinic return.

A commercially available software package (SPSS 21.0 for Windows Evaluation version) was used for statistical analysis. Numerical data are expressed as the means ± standard deviations or medians with interquartile ranges and compared using Mann–Whitney U test. Nominal data are expressed as numbers with percentage and compared using Pearson’s Chi-square exact test. A *p* value less than 0.05 was considered statistically significant.

## 3. Results

**Figure 2:**
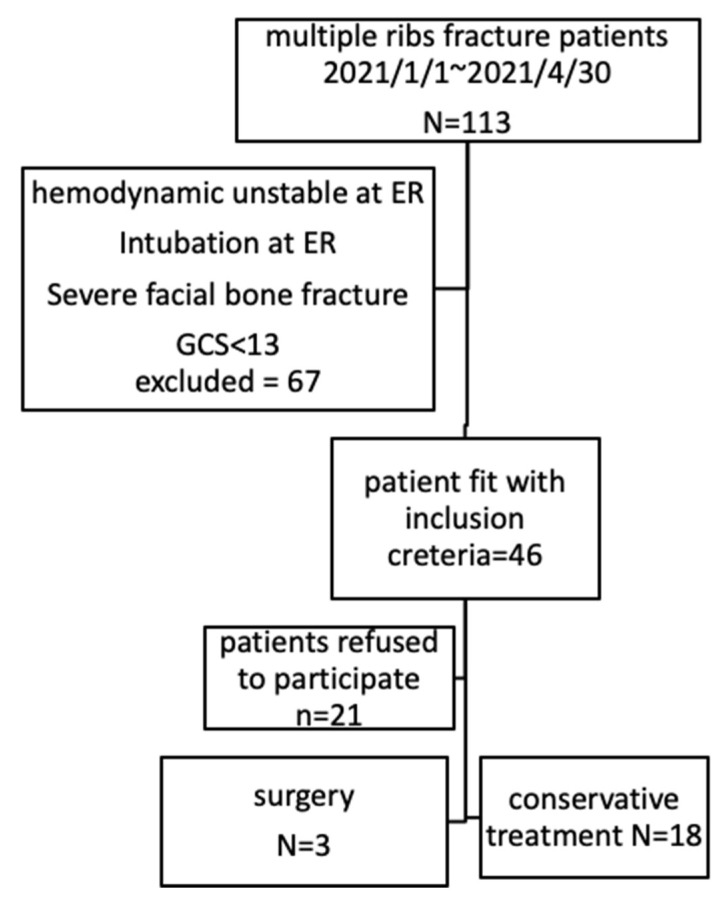
The flowchart of this study.

During this period, there were 113 patients suffering from multiple rib fractures who were in our hospital. We excluded 67 patients because of hemodynamic instability and could not perform spirometry because of facial bone fracture, intubation, or unclear consciousness. There were 25 patients who refused to participate because they refused to follow up in our hospital. We included 21 patients who could perform the Restart system after the introduction as Figure 2 presented.

The initial respiratory parameters and the app usage satisfaction were appropriate and above the normal level. The steps include: waking up the device, launching the app, information input, beginning respiration with a spirometry, review and retake or acceptance of captured data, question response, and submission. There was no difficulty in performing most steps except beginning respiration with the spirometry (competence rate 63%).

**Figure 3:**
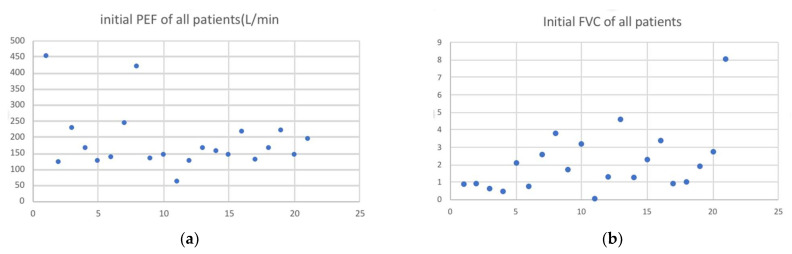
(**a**) The initial peek expiratory flow (PEF) of all patients; (**b**) initial forced vital capacity (FVC) of all patients.

The demographic characteristics of these participating patients are shown in Table 1. The average age is 59 ± 6 years old, the median ISS score is 17 ± 8, and male dominance is noted (17 versus 4). The median rib fractures number is 5 ± 1 with 14 patients having segmental fracture but only 1 with flail chest. The patient with flail chest also accounted for our only intubation 2 days after his admission due to progressive respiratory failure and surgical fixation was also arranged for him on the 4th day of hospitalization. An total of 10 of these patients had hemothorax, 9 of them had pneumothorax, but only 7 of them required chest tube insertion. The median length of stay in hospital is 6 ± 3.5 with three patients admitted to ICU during the hospital course. No mortality case was noted, but two pneumonia cases occurred in these patients. Eight of these patients have residual pleural effusion more than 6 weeks after initial trauma insult as Table 1 presented. The initial PEF and FVC of these patients are shown in Figure 3a,b; the median of PEF is 170 L/min and 1.8 L in FVC.

We further divided the patients into two groups according to our previous compliance criteria as Table 2.

**Figure 4:**
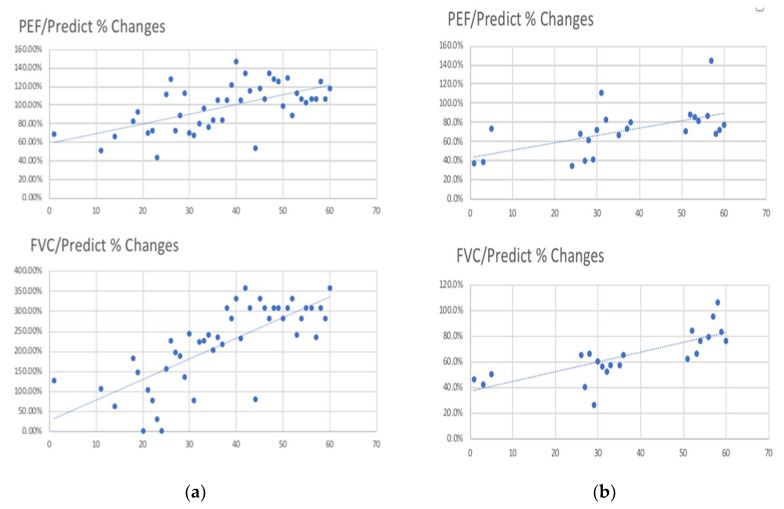
The illustration of lung function parameters monitoring in (**a**) the good compliance and (**b**) poor compliance patients.

There were 10 patients in the good compliance group and 11 in the poor compliance group. There are no differences between these two groups in gender, age, ISS score, trauma mechanism, or the morphologic pattern of the chest trauma (the presence of flail chest or not and the number of fractured ribs). Invasive procedures such as surgical intervention or chest tube insertion are also comparable in both groups. The only difference between them was the BMI. It was 26.1 in the good compliance group versus 23.5 in the poor group. The initial PEF is much better in the good compliance group (195.8 versus 146, *p* = 0.035). However, the improvement of PEF is not significant. Instead, we found that although the initial FVC was without difference between these two groups, the improvement of FVC in 4 weeks is better in a good compliance group (110%), with a poor compliance group (21%) with a significant difference (*p* = 0.049). We also identified the rate of presence of pleural effusion in 6 weeks is lower in the good compliance group (10%) than the poor compliance group (66.7%) with significance (*p* = 0.017). There were two cases who developed pneumonia during the hospitalization. However, it is not statistically significant that they all belonged to the poor compliance group. We calculated every patient’s improvement of lung function including PEF and FVC and made the improvement percentage slope for every patient. Figure 4a presented the example of the PFT parameter of the good compliance patient and Figure 4b showed the PFT of the poor compliance group. Every dot means each lung function they perform; we can see that the slope of the good compliance group is much steeper. Moreover, we found the readmission rate in 90 days is lower in the good compliance group (10%) compared with the poor compliance group (27.3%, *p* = 0.586).

## 4. Discussion

In this study, we presented the feasibility of remote technology that can assist the chest trauma patient to rehabilitate their injured chest with digitized recording. With the intelligent spirometry, the patients can measure and record their daily pulmonary function and recover to normal activity and respiratory status earlier. With continuous monitoring, we can identify the compliance and easy performance of telemedicine in trauma care. The medical descriptions were enhanced, as previous studies presented [19]. In the pandemic era, remote spirometry can be a substitution of standard spirometry to monitor the respiratory condition at home. Furthermore, in this study we identified the compliance of telemedicine as another key to impact a patient’s outcome. With high compliance with the Restart system the patients have better FVC, and less residual pleural effusion rate compared with poor compliance patients.

It has been widely discussed about how to evaluate the severity and outcome of blunt chest trauma physiologically. Spirometry had been proposed as a good measurement tool and is also considered more objectively compared to pain score [20]. For geriatric patients, the spirometry data gained in the first 24 h after trauma can be used as a predictor for discharging home or to a rehabilitation institution [21]. However, traditionally it causes more pain when patients leave their ward to the lung function room for spirometry. Additionally, it is not practical to request the patient’s return for a regular PFT. Furthermore, there were several ways to replace lung function as an indirect tool for lung function evaluation, including the measurement of movement of the chest wall or questionnaire for breathing pattern [4,22]. In the era of a pandemic, the centralized facility will increase the possibility of spread of viral infection. The high possibility of airdrop spread of virus is another consideration in current respiratory procedure and rehabilitation [23]. Remote devices with telemedicine or telerehabilitation became the alternative options to maintain the minimal requirement of the post-discharge follow up [24,25,26]. Numerous apps and studies showed their function to record, and also presented the post-op pain scale by apps in mobile devices. These can replace the pen writing based records which helps to prevent the bias of recall and saves time for physicians to review the summary from patients during visits [19]. In this study, we incorporate pulmonary function and pain scale into a single recording app which saves the caregiver manual work and time. It also increased the convenience during operation. The user-based operating system offered additional benefits.

Incentive spirometry has also been pointed out as a replacement for standard spirometry because it can be used at bedside or after discharge [27]. Some studies found that incentive spirometry can be effective in both lung rehabilitation and guidance of treatment choice [28,29]. However, the information from incentive spirometry is still much less than that of traditional spirometry, and it is difficult to record the pulmonary functional parameters objectively. With this mobile spirometry and telemedicine, patients can easily perform PFT without frequency limitation. Compared to incentive spirometry, patients can also read their own data immediately. With definite lung function, this system can provide a reward mechanism that encourages patients to perform spirometry more times making it a tool for rehabilitation. It can also be brought home with patients as a consecutive and long-term lung function observation system.

The compliance of medical advice and description is critical for rib fracture care, especially when patients return home. In the past, we did not have good methods and materials to monitor patients’ pulmonary rehabilitation at home. With the assistance of the Restart system, we can monitor patients’ PFT and compliance regularly and remotely. In this study, we found the patients with good compliance have significant improvement of FVC compared with poor compliance patients. The improvement of FEV1 was also higher in good compliance patients, although there was no significant difference. Regular performance of lung expansion exercise and pulmonary rehabilitation showed impact on final PFT parameters in rib fractures patients [8,30]. Additionally, the frequency of using this spirometry system has positive effects on patients’ rehabilitation. The good compliance also reduces the risk of presence of residual pleural effusion 8 weeks after rib fracture. One of the advantages of telemedicine is to increase the compliance of medical descriptions. Our study also supports this perspective in chest trauma patients. Since decreasing the possibility of pulmonary complication, we found good compliance patients have less emergency visits or readmission (10%), compared with poor compliance groups (27.3%), which supports the impact of the Restart system on multiple rib fractures patients.

There were several studies for the role of surgical intervention in rib fractures. Flail chest is first proved beneficial in surgical intervention [31,32,33], then it was promoted in multiple rib fractures [33,34,35,36]. The benefit is not restricted medically but also socially. Health care use and total cost is much lower in the surgical group than the non-operative group [37]. In our study, there were three patients who accepted internal fixation surgery for rib fractures. We found that all patients with surgical intervention had great improvement after the operation. Compared to non-operative treatment patients, their lung function parameters maintained a steady result in the first week of surgery. In a previous study, geriatric multiple rib fractures patients would have significant lung function improved 5 days after internal fixation [38]. The other study also pointed out that a better spirometry outcome can predict earlier discharging and better clinical outcome [21]. Although only three patients in our study had accepted surgical intervention, the final clinical outcome was satisfactory. Their compliance with this remote system was adequate, and their clinical outcome was too. The improvement, although not statistically significant, was apparent after surgical intervention. The remote spirometry system clearly pointed out the lung function change is crucial between surgical and non-surgical patients. However, we did not find the difference between flail chest or multiple rib fractures like one other study found [39]. The possible reason could be the relatively small number of patients in our research.

Compared to the young group, we identified that the older group also performed well with this system. Age is not a determinant factor in compliance of this system. The easy usage and simple application design determine the possibility of all the trauma patients included into digital treatment. The study provided valuable information about the feasibility and adequacy of an internet-based intervention for the management of chest rehabilitation of rib fractures, which can be used to guide subsequent research.

### Limitation

This study showed the brilliant impact that telemedicine and mobile spirometry can have in helping patients with rib fractures. However, several limitations should be considered when interpreting the results. First, all assessments were conducted online, and inclusion relied exclusively on self-reported data; therefore, the internal validity and generalizability to a larger clinical population might have been compromised. Second, the sample size was limited, and the sample did not represent all trauma patients. Therefore, subsequent research should investigate the efficacy and cost-effectiveness of the mobile spirometry and remote app in a fully powered and large randomized control trial. Third, compliance improvement is another consideration to test the efficacy of telemedicine; poor compliance occurred in almost half of our patients. Since this is not a standard medical performance, we do not have the authority to command patients to do this, which might be another limitation of digital health devices in further application. Good user feedback and user experience interface should be input to increase the compliance of this system. Finally, the last limitation will be the cyber threat that exists in telemedicine. More efforts will be done with the cooperation of engineers for complete protection of patients’ privacy.

## 5. Conclusions

The patients were enthusiastic about partnering with their health providers in novel ways to optimize their healthcare. In this study, we present mobile spirometry with a remote app to improve a patient’s post-discharge respiratory rehabilitation and more trustworthy monitoring of their pulmonary function. A patient with multiple rib fractures is under significant risks of respiratory complication, and the respiratory rehabilitation should constantly perform given that we are breathing in every second. This telemedicine system gives us better feedback between patients and physicians.

Moreover, the results were positive that improvements were found in both functional (better lung function outcome) and anatomical (less residual pleural effusion) aspects. Although mHealth will certainly not replace physician contact, it will serve as a digital assistant for diagnostic, therapeutic, and follow-up purposes for supporting patient recovery. This is especially important in the pandemic era, now that face-to-face contact is limited. Using this telemedicine system, we can monitor patients’ lung function and rehabilitation remotely and that can be extended in acute care and personalized medicine fields considering that we are facing the threat of pandemics right now and potentially in the future. Furthermore, it will be a promising tool for a complete caring system.

## Figures and Tables

**Table 1:** The demographic characteristics of multiple rib fractures patients.

	N = 21
Age (years)	59 ± 6
Male gender (*n*, %)	17 (80.9%)
BMI (Mean ± SD)	25 ± 2.3
Rib fracture number (Median ± IQR)	5 ± 1
Flail chest (*n*, %)	1
Segmental fracture (*n*, %)	14
ISS (Mean ±SD)	17 ± 8
Hemothorax (*n*, %)	10 (47.6%)
Pneumothorax (*n*, %)	9 (42.9%)
Chest tube (*n*, %)	7 (33%)
Length of stay days (Median ± IQR)	6 ± 3.5
ICU admission rate (*n*, %)	3 (14%)
Length of ICU stay days (Median ± IQR)	4 ± 0.5
Complication (*n*, %)	7 (33%)
Pneumonia (*n*, %)	2 (9.5%)
Persist pleural effusion >6 weeks (*n*, %)	7 (33%)
Mortality (*n*, %)	0 (0%)

BMI: Body mass index; SD: standard deviation; IQR: interquadral range; ISS: Injury severity scale; ICU: intensive care unit.

**Table 2:** The comparison of good and poor compliance group.

	Good Compliance N = 10	Poor Compliance N = 11	*p* Value
Age (years, median, IQR)	57 (3.4)	60 (8.5)	0.459
Male gender (*n*, %)	9, 90%	8, 72.7%	0.586
BMI (median IQR)	26.1 (0.5)	23.5 (1.8)	0.029 *
ISS (median IQR)	16.5 (4.5)	18 (9)	0.395
Associate injury			
Head injury (*n*, %)	0, 0%	4, 36.4%	0.09
Solid organ injury (*n*, %)	1, 10%	0, 0%	0.476
Pelvic fracture (*n*, %)	1, 10%	1, 9.1%	1
Number of rib fractures (median IQR)	4 (0.8)	5 (0.5)	0.234
Flail chest (*n*, %)	1, 10%	0, 0%	0.361
Hemothorax (*n*, %)	6, 60%	4, 36.4%	0.395
Pneumothorax (*n*, %)	6, 60%	3, 27.3%	0.198
Chest tube insertion (*n*, %)	4, 40%	3, 37.3%	0.659
Initial FVC (median IQR)	1 (0.04)	1.29 (0.06)	0.223
Improvement of FVC (%)	110% (66)	21 (18)	0.049 *
Initial FEV1 (median IQR)	195.82 (67)	146.7 (2.5)	0.035 *
Improvement of FEV1 (%)	64 (5)	36 (18)	0.888
Length of stay (days, median IQR)	6 (2.8)	8 (3.5)	0.671
Surgical intervention (*n*, %)	3, 30%	0, 0%	0.09
Complications	1, 10%	7, 66.7%	0.017 *
Pneumonia	0, 0%	2, 18.2%	0.262
Presence of Pleural effusion > 6 weeks (*n*, %)	1, 10%	7, 66.7%	0.017 *
Readmission in 90 days (*n*, %)	1, 10%	3, 27.3%	0.586

BMI: Body mass index; SD: standard deviation; IQR: interquadral range; ISS: Injury severity score; ICU: intensive care unit; FVC: Forced vital capacity; FEV1: Forced expiratory volume. *: Statistically significance.

## Data Availability

The data is available after request the corresponding author for research purpose.
